# Screening Health-Related Social Needs in Hospitals: A Systematic Review of Health Care Professional and Patient Perspectives

**DOI:** 10.1089/pop.2022.0279

**Published:** 2023-06-01

**Authors:** Ricardo J. Trochez, Sahana Sharma, Deonni P. Stolldorf, Amanda S. Mixon, Laurie L. Novak, Amol Rajmane, Irene Dankwa-Mullan, Sunil Kripalani

**Affiliations:** ^1^ Center for Health Services Research, Vanderbilt University Medical Center, Nashville, Tennessee, USA.; ^2^ School of Nursing, Vanderbilt University, Nashville, Tennessee, USA.; ^3^ Department of Medicine, Vanderbilt University Medical Center, Nashville, Tennessee, USA.; ^4^ Department of Biomedical Informatics, Vanderbilt University Medical Center, Nashville, Tennessee, USA.; ^5^ IBM Watson Health, Cambridge, Massachusetts, USA.

**Keywords:** social determinants of health, health-related social needs, screening, attitudes

## Abstract

Health outcomes are markedly influenced by health-related social needs (HRSN) such as food insecurity and housing instability. Under new Joint Commission requirements, hospitals have recently increased attention to HRSN to reduce health disparities. To evaluate prevailing attitudes and guide hospital efforts, the authors conducted a systematic review to describe patients' and health care providers' perceptions related to screening for and addressing patients' HRSN in US hospitals. Articles were identified through PubMed and by expert recommendations, and synthesized by relevance of findings and basic study characteristics. The review included 22 articles, which showed that most health care providers believed that unmet social needs impact health and that screening for HRSN should be a standard part of hospital care. Notable differences existed between perceived importance of HRSN and actual screening rates, however. Patients reported high receptiveness to screening in hospital encounters, but cautioned to avoid stigmatization and protect privacy when screening. Limited knowledge of resources available, lack of time, and lack of actual resources were the most frequently reported barriers to screening for HRSN. Hospital efforts to screen and address HRSN will likely be facilitated by stakeholders' positive perceptions, but common barriers to screening and referral will need to be addressed to effectively scale up efforts and impact health disparities.

## Introduction

Nearly three fourths of patient health outcomes is determined by a complex interplay of behavioral, health care, and social factors.^1^ In efforts to improve whole-person care, health care professionals and hospital systems in the United States have increased attention to assess and address health-related social needs (HRSN) such as food insecurity, housing instability, and transportation needs in clinical settings.^2–4^ Despite substantial attention in community and other outpatient settings, comparatively little research has explored HRSN within inpatient contexts.

National scientific groups have called for urgent attention to systematically address HRSN in health care to reduce and eliminate health disparities.^5^ Hospitals have responded to this urgent call with 917 US hospitals committing about $2.5 billion toward interventions addressing social needs.^6^ Furthermore, robust information systems (eg, electronic health records [EHRs]) are being developed with the integration of social needs screening tools.^7–10^ Notwithstanding, health care professionals across different care settings (ie, acute care, outpatient care) continue to perceive screening for HRSN as challenging and complex to adopt, citing barriers such as lack of resource awareness, adequate training of staff, and adjustment of workflows and role responsibilities.^11^

Most published research and implementation guidance about social needs screening is based in the outpatient setting.^11,12^ Comparatively little focus has been given to assessment of HRSN in the inpatient environment. Yet, with the extensive visibility and influence of hospitals in communities, they are uniquely positioned to screen for social needs and care for or refer to address them.^13^

Moreover, new 2023 Joint Commission standards (LD.04.03.08) related to reducing health care disparities now require hospitals to assess patients' HRSN and provide information about community resources and support services.^14^ (Joint Commission explains preference for the term HRSN instead of social determinants of health (SDOH) “to emphasize that HRSNs are a proximate cause of poor health outcomes for individual patients as opposed to SDOH, which is a term better suited for describing populations.”^14^) The goal of this systematic review was to better understand prevailing attitudes among provider and patient stakeholders about screening for HRSN in US hospitals. Furthermore, the review sought to understand common barriers to help inform implementation and advancement of screening initiatives in adult inpatient settings.

## Methods

### Database search and article identification

**FIG. 1.**
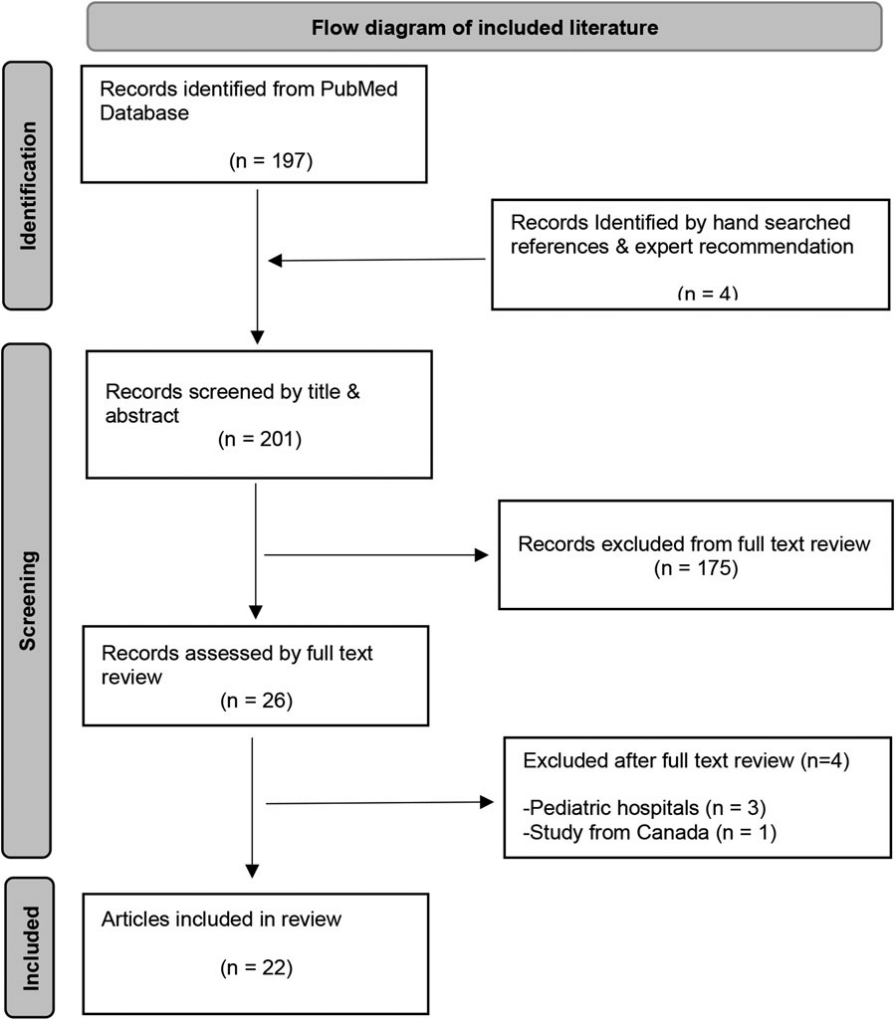
Flow diagram of included literature.

This literature synthesis encompasses health care professionals' perceptions, patients' perceptions, and barriers and facilitators to screening and use of HRSN in US hospitals. The authors conducted an online literature search guided by a medical librarian and in accordance with the Preferred Reporting Items for Systematic Reviews and Meta-Analyses (PRISMA) guidelines. A PubMed search was conducted in August 2021 and updated in January 2022, using a combination of keywords and Medical Subject Heading “MESH” terms related to SDOH, social risks, or social needs, in clinical settings. The authors also contacted experts in the field to recommend articles, searched a national social determinants of health bibliography (SIREN), online-searched conference posters of a 2020 national meeting, and hand-searched references of selected articles (Fig. 1).

### Literature selection and criteria

Original research studies were included if they took place in US-based adult hospital or health system settings, reported perceptions of health care professionals or patients on screening for or using patients' HRSN data, or reported barriers and facilitators relevant to hospital-based HRSN screening. Articles reporting results exclusively from pediatric settings were excluded, as well as those not available in English. No exclusion criteria were applied to study design, sample size, or date of publication.

### Data extraction and synthesis

Two reviewers (R.J.T. and S.S.) independently screened all articles by title and abstract, referring to full-text articles when needed. They (R.J.T. and S.S.) met 2 times to resolve discrepancies through discussion and reached consensus under the supervision and input of a senior researcher (S.K.). Data extraction was completed by R.J.T. and S.S., including each article's basic study characteristics such as authors, sample size, assessment tools used, respondent type, and setting/US region. R.J.T. independently further cross reviewed and organized the main findings from literature by research methodology—quantitative, mixed methods, and qualitative. Quantitative outcomes extracted were primarily frequencies related to survey question responses. Given the varied composition of the studies, including qualitative and mixed-methods studies, the authors did not conduct a meta-analysis, nor a formal assessment of risk of bias given their observational design.

## Results

Studies of hospitals included in this systematic review span several US regions, with diversity observed in clinical settings, methods, and sample composition. The search identified 22 studies meeting selection criteria, including 13 quantitative, 3 qualitative, and 6 mixed-methods studies (Table 1). All 13 quantitative studies reported results with proportions and/or odds ratios and corresponding *P* values. Mixed-methods and qualitative studies reported findings mostly from emerging themes analyses and quotes (Table 2). Study sample sizes ranged from 10 to 2018 research participants for 20 studies. Two studies used administrative data sets, with one containing 93,606 patients, and another one >13 million patients. All articles were published between 2014 and 2021.

**Table 1.** Characteristics of Included Studies

Article no.	Refs.	Study design	Assessment tool used	Sample size (N)	Participants	Setting/US region
1	Bleacher et al^17^	Prospective cohort	11-Item survey, locally developed	2018 Patients, 45 providers	Patients and providers	AMC; Mid-west
2	Bensken et al^35^	Retrospective cohort	ICD-10 and z-codes in national data set	13+ Million patients	Patients	Multiple sites
3	Cottrell et al^34^	Retrospective cohort	15-Item survey based on PREPARE	31,549 Patients	Patients	Health information network; West Coast
4	Navathe et al^36^	Retrospective cohort	EHR, administrative data sets	93,606 Patients	Physician notes, patients EHR	AMC; Northeast
5	Schickedanz et al^16^	Cross-sectional	21-Item survey locally developed	258	Providers	Integrated health system; West Coast
6	Fraze et al^22^	Cross-sectional	National Survey of Healthcare Organizations and Systems	739 Hospitals, 2190 practices	Varied: Directors, managers	Multiple sites
7	Losonczy et al^18^	Cross-sectional	40-Item survey locally developed	432	ED physicians	Multiple sites
8	Phillips et al^31^	Cross-sectional	71-Item survey adapted from Persaud 2018	768	Providers	AMC; Mid-west
9	Purnell et al^24^	Cross-sectional	3 Validated surveys (Weissman 2005; Paez 2008)	1220	Physicians	AMC; Northeast
10	Rogers et al^30^	Cross-sectional	8-Item survey locally developed	1161	Patients	Multiple sites
11	Sand-Jecklin et al^19^	Cross-sectional	Brief Health Literacy Screen (patients), 10-item survey locally developed	25,557 patients, 119 nurses	Patients, nurses	AMC: Southeast
12	Wahab et al^20^	Cross-sectional	REALM-8 (patients) 27-item survey locally developed (providers)	113 Pairs	Patients and providers	AMC; Mid-west
13	Zettler et al^32^	Cross-sectional	10-item survey locally developed	165	Providers	Multiple sites
14	Cartier and Gottlieb^21^	Mixed methods	Multiple surveys, interviews	Multiple, 95–7002	Patients and providers	Multiple sites
15	Freibott et al^27^	Mixed methods	PRAPARE survey, staff interviews	692 Patients, 8 care staff	Patients and providers	AMC; Northeast
16	Hamity et al^23^	Mixed methods	Surveys/interviews, focus groups	68 Patients, 90 clinicians	Patients and providers	Integrated health system; multiple US regions
17	Kostelanetz et al^15^	Mixed methods	21-Item survey adapted from Schickedanz 2019	193	Providers	AMC; South
18	Norton et al^29^	Mixed methods	Survey from OCHIN	53	Patients	AMC; Northeast
19	Wallace et al^25^	Mixed methods	10-item survey developed locally, interviews, focus groups	2821 Patients, 10 staff	Patients and providers	AMC; West
20	Dauner and Loomer^33^	Qualitative	Interviews, focus groups	37	Providers	Multiple sites
21	Drake et al^28^	Qualitative	Interviews, focus groups	10 Patients, 5 case managers	Patients and case managers	AMC; Southeast
22	Powell et al^26^	Qualitative	Focus groups, interviews	39	Providers	AMC; Northeast

AMC, academic medical center; ED, emergency department; EHR, electronic health record; ICD-10, International Classification of Diseases, 10^th^ Revision; PREPARE, Protocol for Responding to and Assessing Patients' Assets, Risks, and Experiences; SDOH, social determinants of health.

**Table 2.** Literature Synthesis and Main Findings

Article no.	Refs.	Main findings	Research method
1	Bleacher et al^17^	Screening program undersampled African Americans, oversampled Caucasians. 91% of staff and 92% of clinicians agreed that screening benefitted patients. 96% of clinicians agreed to continue screening for HRSN despite the additional work. Clinicians were already involved with screening intervention program.	Quantitative
2	Bensken et al^35^	Used z-codes to identify HRSN in large national readmissions database. Suggest varying use of z-codes within institutions to document HRSN. Housing and employment emerged as 2 most commonly documented factors. Patients coded in 5 domains had higher readmission rates than those in only 1 domain.	
3	Cottrell et al^34^	More than half of screening included responses only from 1 domain. About 50% of screenings reported came from only 4 sites, out of 106 sites. Patients with incompletely filled survey counted as screened. Screening tools availability does not automatically lead to use.	
4	Navathe et al^36^	Prevalence of social factor in ICD-9 codes plus EHR and MD notes (tobacco use 30%, alcohol use- ∼15%, housing instability <5%, poor social support ∼15%). Physician notes reflected social needs more than ICD-9 codes in patient EHR. Poor social support and housing instability significantly associated with increased readmission risk.	
5	Schickedanz et al^16^	84% support screening for HRSN in clinical settings. 93% and 95% agree that it can improve trust and overall care, respectively. 23% only actually screen patients for social needs always. Differences by health profession toward perceived barriers.	
6	Fraze et al^22^	24% of hospitals sampled screened for all 5 social needs versus 15% physician practices. Only 8% of hospitals reported no screening, compared with 33% in physician practices. Interpersonal violence was the most common social risk screened for in hospitals (75%). Academic medical centers more likely to screen for HRSN compared with other hospitals, 49% versus 23%.	
7	Losonczy et al^18^	Number of doctors who routinely ask about social needs range from 61% to 100%. 80% of doctors reported they would like more resources. 70% reported they would attend educational sessions if available.	
8	Phillips et al^31^	50% reported feeling more confident in ability to discuss access to care issues compared with other HRSN. Barriers: lack of time to address HRSN, unfamiliarity of internal/external resources. Reported need for interdisciplinary education and collaboration.	
9	Purnell et al^24^	Providers who reported moderate/major structural problems more likely to report low skillfulness how to address HRSN, OR 3.2, *P* < 0.01. 45% reported poor access to written materials in other languages. 21% reported poor access to interpreters. <50% of 1220 clinicians engage in behaviors to address barriers to HRSN >75% of the time.	
10	Rogers et al^30^	69% of patients agreed social needs impact health. 85% responded that health system should ask about social needs. 88% patients reported that health system should help address HRSN. Significant differences observed by race, gender, age, education, and HRSN need history. Compared with males, females more likely to assess (OR 1.4, *P* < 0.05) and address (OR 1.7, *P* < 0.001) social needs.	
11	Sand-Jecklin et al^19^	Nurses indicated positive perceptions of health literacy screening implementation in hospital. No significant difference in feasibility scores by years of experience, or age groups. 20% of screened patients were identified as at risk for health literacy limitations.	
12	Wahab et al^20^	Residents identified correctly 97% of patients who were not at risk for low health literacy. Identified correctly only 12.5% of those who were at risk for low health literacy. Residents' knowledge pre- or post-education did not improve. Resident physicians overestimate patient health literacy and its implications to patient care interaction.	
13	Zettler et al^32^	Main HRSN barriers: Physicians asking patients about HRSN interfering with their care (18% all the time, 51% often, and 29% occasionally). Majority of physicians noted time constraints for assisting patients with social needs (34% strongly agree and 47% agree). Majority agreed programs to assist with social needs not readily available (20% strongly agree and 56% agree).	
14	Cartier and Gottlieb^21^	15%–100% of respondents agreed their organization screens for at least 1 HRSN. For hospitals, results ranged for screening between 62% and 91%. 21 of 23 surveys did not provide a denominator for total population served.	Mixed methods
15	Freibott et al^27^	66% reported food, transportation, and housing needs. Lack of standardized referral process made screening unsustainable or unjustifiable. 4 staff reported screening optimizes health care delivery and outcomes. All staff interviewed reported screening tool was short, enhanced ease of use. Some patients were reluctant to report needs.	
16	Hamity et al^23^	Members/patients and clinicians agreed social needs impact health. Providers were on average not screening for HRSN, yet believed screening may improve trust. Members/patients agreed health system should help address social needs. Both groups reported importance of social needs assessments that leads to actionable information. Both groups reported importance of delineating who should do social needs assessments.	
17	Kostelanetz et al^15^	94% reported HRSN data could be used to improve patient care. 91% and 93% agreed that it could improve trust and communication, respectively. Differences in perceived importance versus actual screening for housing instability, 73% versus 53%. 51% of providers cited lack of resources is biggest barrier to address HRSN. 45% and 33% reported lack of time and support staff as barriers, respectively.	
18	Norton et al^29^	>50% patients had positive perceptions about screening process. 47% of patients screened positive reported inability to connect with resource/help. 40% of patients with positive screening were hard to reach by phone. 25% of patients declined services/help offered after positive screening.	
19	Wallace et al^25^	7% of patients completed the process from screening to referral with community resource. ED staff communicated discomfort expanding roles, questioned usefulness of screening. Patients communicated desire for improved understanding of their social needs. Older male non-White and Hispanic patients were more likely to complete referral process.	
20	Dauner and Loomer^33^	Screening varies by time and clinician. Lack of access to internet, lack of labor, financial, and social services were screening barriers. Occurs informally between inpatient and outpatient settings Lack of systematic process to follow up on referrals also cited as barrier to screening.	Qualitative
21	Drake et al^28^	Clear communication, proactive initiative, and nonjudgmental attitude valued by patients. Patients shared negative experiences related to discrimination in health care when seeking assistance. Screening completed in <10 min. Patients were receptive to sharing information on HRSN.	
22	Powell et al^26^	Participants reported feeling unable to motivate patients to follow-up after discharge in the setting of substance abuse or mental health struggles. Providers perceived patients distrust in health care system affects screening efforts. Suggestion improving health system visibility in community. Suggestion to increase number of minority providers and staff.	

HRSN, health-related social needs; ICD-9, International Classification of Diseases, 9^th^ Revision; OR, odds ratio.

### Provider perceptions of social risks screening

There were 6 studies reporting quantitative data from surveys about positive provider perceptions on screening for patients' HRSN. Four out of these 6 studies reported differences between health care professionals' perceived importance about screening and actual screening rates at the same institution. One study found that, among a sample of 193 participating health care professionals, 94% believed that HRSN screening could be used to improve patient care, whereas 91% agreed that screening could improve trust of providers by patients, and 93% that screening could improve communication.^15^

Yet, notable differences between perceived importance and actual screening rates were found, for example, on housing instability (73% rated as important vs. 53% actually screened). Similarly, another study reported that 84% of health professional survey respondents agreed that HRSN screening should be a standard part of care, as it can improve trust (93%), communication with patients (96%), and overall care (95%).^16^ However, in this study only 23% of clinicians reported that they always screened for patients' social needs.

Bleacher et al found that 92% of clinicians and 91% of staff surveyed agreed that screening for social needs benefited patients, and 96% of the providers agreed to continue screening despite the additional workload.^17^ A study assessing ED physicians' perceptions also reported variation in routine screening for any specific HRSN (61%–100%).^18^ Notably, in this study, 80% of ED physicians reported that they would like more resources to screen and refer patients for assistance, and 70% that they would attend educational sessions if they were available. Two quantitative studies focused on health literacy screening; one study found that nurses held positive perceptions for implementing screening for health literacy during hospital admissions, regardless of years of nursing experience or age.^19^ The other study reported that resident physicians overestimate patients' health literacy and may underestimate the influence of low health literacy on patients' understanding during clinical interactions.^20^

Four additional studies reported that actual HRSN screening also varies widely between institutions. One multisite study found the variation in health care professionals' reported screening for at least 1 social need to range between 62% and 91% among hospitals.^21^ A similar study of 739 hospitals found that academic medical centers were more likely to screen for HRSN compared with other hospitals (49% vs. 23%).^22^ Another multisite study nested within a large integrated health care system in the Western United States found that clinicians were regularly not screening for HRSN, despite thinking that assessing patients' social needs was a valuable opportunity for gaining actionable information.^23^ In comparison, another study within a large health care system in the Eastern United States found that less than half of 1220 physicians surveyed reported engaging in behaviors to address cultural and social factors more than 75% of the time.^24^

The 4 included qualitative studies identified additional provider concerns about screening HRSN. One study reported that frontline staff had feelings of discomfort and questioned usefulness of screening when assessing HRSN.^25^ Another study found that staff felt unable to effectively motivate patients to pursue follow-up or assistance after being discharged from hospital admission related to substance abuse or mental health struggles.^26^ Providers also perceived patient distrust in the health care system affecting screening efforts.^26^ Similarly, 1 study reported that a lack of standardized referral processes made patient screening difficult to justify or sustain, and that patients could be reluctant to reveal social needs despite screening tools being easy to use.^27^

### Patient perceptions of social needs screening

Four studies on patients' perceptions of social needs screening reported positive attitudes more often than not. One study found that patients were overall receptive to sharing information on HRSN and that they valued clear communication and a nonjudgmental attitude during screening.^28^ However, some patients worried about questions targeting or profiling their low-income status. These patients recounted negative personal experiences of racial and ethnic discrimination when seeking assistance in health care.^28^ Another study found that more than half of participants had positive feedback about the social needs screening and assistance process, mentioning that they liked that people were trying to help them.^29^ However, 25% of patients who screened positive for HRSN declined services when offered or said help was no longer needed, and 40% of patients with a positive screen reported an inability to connect with resources or assistance.^29^

In another study from a large integrated health system, patients agreed that their health system should ask about (85%) and help address (88%) HRSN, and that social needs impact health (69%).^30^ However, significant differences in perceptions were found by social needs history, gender, race, age, ethnicity, and education.^30^ Specifically, patients were 10 times more likely to agree social needs impact health if they had experienced HRSN within the past year. Moreover, women were 40% and 70% more likely to support assessing and addressing HRSN compared with men, respectively.

Patients aged 41–60 were more likely to agree their health system should dedicate financial resources to address HRSN compared with those <41 years of age. Surprisingly, racial/ethnic minority patients including Hispanic, Black, or Asian/Pacific Islander were less likely to perceive that social needs impact health compared with non-Hispanic Whites. Furthermore, college graduates were almost 2 times more likely to believe social needs impact health.^30^ Another study reported that patients desired an improved understanding of their HRSN, despite their concerns about stigmatization and privacy, which further underscore the influence of to whom, what, and how information is given or collected.^25^

### Barriers to social needs screening and data use

The search identified 12 studies that reported barriers related to screening for HRSN. Lack of knowledge or awareness about resources available, lack of training and support, and time constraints were most frequently cited. One study found that 51% of participating providers reported the lack of resources to address patients' social needs as the biggest barrier to screening, followed by the lack of time (45%), support staff (33%), and training to respond to patients' social risks (28%).^15^ Other studies also found that insufficient time to address identified HRSN and unfamiliarity with internal or external resources were barriers to screening.^16,18,31,32^ In another study, difficulty reaching patients by phone for follow-up on social needs and assistance was an important barrier to program implementation, particularly among Spanish-speaking or low-income groups.^29^ In this study, 40% of patients with HRSN were difficult to reach by phone.^29^

In a qualitative study, rural providers described the lack of financial, labor, internet, and community-based social services as barriers to being able to assist patients with social needs.^33^ The authors also mentioned the importance of leadership, collaboration between hospitals, as well as with community agencies, as facilitators to HRSN screening and referral. In another study, health care professionals perceived patients do not trust the health care system or trust that health care providers will be motivated to know their life situations, which hamper efforts to screen for HRSN and other adverse circumstances.^26^

Data quality issues may also be a barrier to effectively screen for HRSN. One study found that patients were counted as being screened even if they only completed 1 domain of the questionnaire.^34^ Another study reported an undersampling of African Americans from those who were eligible for screening.^17^ In contrast, other studies pointed to the use of *z*-codes and International Classification of Diseases, 9^th^ Revision (ICD-9) codes in health care documentation as a possible facilitator of accurately assessing and reporting patients' social needs at both the patient and population level.^35,36^

One study found significant associations between readmission rates and documented social needs by *z*-codes in an EHR, with the caveat that *z*-codes may be under-reported given they are currently not billable and not well known to providers.^33^ Similarly, another study found that identifying aggregate social needs improved notably by combining doctors' notes in the EHR and ICD-9 codes compared with using only ICD-9 codes, consequently yielding an improved estimate of social needs prevalence for the patient population.^34^

## Discussion

This systematic review found that health care professionals and patients predominantly view screening for patients' HRSN as positive. Health care professionals in hospital settings overwhelmingly felt that social needs data are helpful for patient care, and patients are receptive to providing these data. Health care providers also indicated that screening for social needs is an important mechanism for improving patients' trust and enhancing communication with patients. However, HRSN are infrequently collected and used in patient care, which hospitals will need to improve upon to meet new Joint Commission requirements.

Actual implementation of screening lagged perceived importance, likely due to multiple barriers, most often lack of resources, time, and hospitals' fragmented connection to community-based resources. Additional barriers include a lack of training on screening for HRSN and a lack of knowledge or awareness of resources once patients screened positive. However, it must be noted that the majority of respondents in published studies were not skilled social workers, who may have reported different perspectives. Nonetheless, addressing these commonly reported barriers could potentially and substantially improve screening rates in hospitals and attention to patients' social needs.

Several concurrent approaches could enhance hospital capacity to screen and address HRSN. Research and practice-based efforts are needed to determine how hospitals can best build collaborative referral networks with resources in the community. Databases of resources are becoming available to health care professionals for referring patients with social needs. Such databases should increase health professionals' awareness of available resources, reduce time spent to identify potential resources, and when integrated into the EHR, provide a more seamless process for community resource referrals.^37^

Moreover, the reported lack of training in use of screening tools by most health professionals should be addressed through development of focused training materials and programs. Such training has been shown in other contexts to increase provider knowledge, confidence, identification of needs, and resource referrals.^38–42^ Including skilled social workers as an integral part of patient care teams will also help galvanize screening and referral efforts. Embracing multiple strategies in a hospital-wide campaign, while engaging relevant stakeholders from health systems and communities, is likely to be more effective than single strategies alone.

Although patients predominantly perceived screening for HRSN as positive, some also reported negative experiences, and efforts to screen for social needs should be designed to avoid unintended consequences. The majority of patients agree that health care professionals should screen and assist with addressing HRSN. However, this screening must be done with a nonjudgmental attitude and clear communication by health professionals. In the context of social needs screening, patients worry about stigmatization and profiling of low-income status and privacy concerns. This suggests that how data are collected and how information on social needs is provided to patients and providers are important factors to consider when screening tools are implemented. Preserving the dignity and privacy of patients are particularly important throughout the screening process.^43,44^

A few limitations to this review are worth noting. First, the authors did not formally appraise the quality of evidence in the included studies, which were predominately surveys. Second, although the search was relatively comprehensive, it primarily identified articles available through PubMed and may have missed other relevant work, including white papers, industry papers, and gray literature. Third, selected papers included hospital settings, but this was not exclusive of other patient care environments; some studies included respondents from both inpatient and outpatient settings, and it was not possible to separate out these groups.

Despite these limitations, this systematic review identified support for more widespread screening of HRSN and provides guidance for implementing such screening in the inpatient setting. In integrating quantitative, qualitative, and mixed-methods research, the authors found positive perceptions toward HRSN screening efforts to improve patient care and equity; yet qualitative findings explained more details of the complexity and resource-dependent pathway from perception to actuality. These findings are complementary and necessary to advance the knowledge base relating to addressing HRSN to improve health outcomes.

As hospital-based screening of HRSN becomes more widespread, research should evaluate both implementation and clinical outcomes. Implementation research should determine which screening approaches are most feasible to implement, acceptable to patients and health care professionals, and effective in identifying HRSN. It will be important to evaluate not only overall screening completion rates, but also rates within vulnerable patient subgroups, who may be harder to reach through broad screening efforts and may require tailored approaches.

Research should evaluate how to best connect patients and families who want assistance with appropriate health system and community resources, which forms of assistance are most helpful for common HRSN such as food insecurity and transportation needs, and close the loop on whether assistance is actually provided. Moving farther downstream, clinical outcomes research is needed to determine the extent to which screening and addressing HRSN impacts patient health and reduces health disparities. Ideally, outcomes studies will include programmatic details (eg, how screening was performed, which HRSN were addressed and how, what resources were needed) and the cost-effectiveness and sustainability of various approaches.

## Conclusion

Health care professionals and patients believe that social needs impact health, and that assessing and addressing HRSN should be a standard part of care in hospital settings. These findings support new Joint Commission requirements to screen for HRSN as a step toward reducing health disparities. However, hospitals must overcome several common barriers to screening in order for efforts to be more widespread and successful.

Steps include aligning with the organizational mission and priorities, allocating resources, training multidisciplinary staff and engaging expertise of social workers, and growing community-based organization collaborations to advance and sustain screening programs. Patients and providers are willing to engage in a concerted effort to assess and address patients' HRSN systematically in health care, on the idea that it could improve health outcomes, health equity, and social justice. Continued research to demonstrate successful models for inpatient HRSN screening and referral, as well as downstream reductions in health disparities, will be important to develop the evidence base and support continued efforts over time.

## *PROSPERO* Registration

This systematic review was not registered due to currently being completed and thus passed the design phase only eligibility. The review protocol is available on request from the authors.
